# Effect of CYP2C19 polymorphisms on antidepressant prescription patterns and treatment emergent mania in bipolar disorder

**DOI:** 10.1038/s41397-022-00294-4

**Published:** 2022-11-04

**Authors:** Erik Joas, Lina Jonsson, Alexander Viktorin, Erik Smedler, Erik Pålsson, Guy M. Goodwin, Mikael Landén

**Affiliations:** 1 Institute of Neuroscience and Physiology, Department of Psychiatry and Neurochemistry, Sahlgrenska Academy, University of Gothenburg, Gothenburg, Sweden; 2 Department of Medical Epidemiology and Biostatistics, Karolinska Institutet, Stockholm, Sweden; 3 Department of Psychiatry, University of Oxford, Warneford Hospital, Oxford, UK

**Keywords:** Genetics research, Outcomes research

## Abstract

Antidepressant medication is used extensively to treat bipolar depression despite uncertain efficacy. The cytochrome P450 (CYP) 2C19 enzyme metabolize several antidepressants, and polymorphisms in the corresponding gene *CYP2C19* influence plasma concentration and hence treatment outcomes in major depressive disorder. Here, we investigate if *CYP2C19* polymorphisms are associated with antidepressant treatment patterns and the risk of mania when antidepressants are used in bipolar disorder. Two single nucleotide polymorphisms (rs4244285 and rs12248560) were used to classify 5019 bipolar disorder patients into *CYP2C19* metabolic phenotypes ranging from poor to ultra-rapid metabolizers. We used Swedish national registry data 2005–2017 on dispensed medications and inpatient care to estimate risks for early-treatment persistence, treatment discontinuation, switching to a new antidepressant medication, and mania within 3 months of treatment initiation in patients treated with citalopram, escitalopram, sertraline, amitriptyline, and clomipramine. Metabolic phenotypes of *CYP2C19* were not robustly associated with the investigated treatment outcomes based on dispense patterns. Slower metabolism was associated with an increased risk of treatment emergent mania for sertraline (hazard ratio [HR] = 1.3, 95% CI = 1.04–1.62, *p* = 0.02) and the tricyclic antidepressants amitriptyline and clomipramine (HR = 1.46, 95% CI = 1.05–2.02, *p* = 0.024). In a large study of the impact of *CYP2C19* metabolic phenotypes on antidepressant treatment of bipolar depression, we found an association between slower CYP2C19 metabolism and higher risk of treatment emergent mania, which is a step towards personalized risk assessments. There were, however, no clear associations with early treatment persistence, treatment discontinuation, and switching to a new antidepressant.

## Introduction

Antidepressants are extensively used to treat bipolar depression and are sometimes used for prolonged periods of time [1, 2]. The use of the antidepressants in bipolar disorder is, however, controversial [3, 4] due to the risk of treatment emergent mania [5] as well as limited data in support of efficacy [3].

Several antidepressant drugs are metabolized by the cytochrome P450 (CYP) 2C19 enzyme, e.g., citalopram, escitalopram, sertraline, amitriptyline, and clomipramine. Genetic variants in the corresponding gene *CYP2C19* are known to affect enzyme function yielding individual differences in drug metabolism capacity from poor to ultra-rapid metabolizers [6, 7].

Variation in *CYP2C19* is associated with plasma concentration of sertraline [8, 9], escitalopram [10, 11], amitriptyline [12], and clomipramine [13]. Indeed, the Clinical Pharmacogenetics Implementation Consortium (CPIC) guidelines, which focuses on major depression and anxiety disorders, suggests that *CYP2C19* genotyping could be used to identify poor metabolizers of sertraline, citalopram, or escitalopram at risk of side effects due to high plasma concentrations, as well as ultra-rapid metabolizer at risk of treatment failure due to low plasma concentrations [6]. Moreover, poor metabolizers treated with tricyclic antidepressants could end up with an unfavorable ratio between parent drug and active metabolites with different pharmacological properties [7]. It has therefore been suggested that tricyclic drugs should be avoided in poor and ultra-rapid metabolizers [6, 7, 14]. Further, other cytochrome P450 enzymes involved in drug metabolism may modify CYP2C19 effects [7, 15].

Data on the impact of *CYP2C19* genotype on antidepressant treatment effects in bipolar depression is scarce [16], but one study suggested that the CYP2C19 poor metabolism phenotype is more prevalent in treatment resistant bipolar disorder compared with treatment resistant major depressive disorder [17].

The aim of the present study was to test the effect of *CYP2C19* metabolic phenotypes when antidepressants metabolized by CYP2C19 (citalopram, escitalopram, sertraline, amitriptyline, and clomipramine) are used by patients with bipolar disorder. To this end, we defined *CYP2C19* metabolic phenotype by the rs4244285 and rs12248560 polymorphisms in a cohort of more than five thousand genotyped patients with bipolar disorder using information on dispensed medication from 2009 to 2017. We first tested if *CYP2C19* metabolic phenotypes are associated with antidepressant response, side effects, and adverse events—as inferred from treatment discontinuation or switching to another antidepressants—in bipolar depression. Second, we tested if *CYP2C19* metabolic phenotypes are associated with antidepressant induced treatment emergent mania, using inpatient diagnoses of mania following treatment initiation of an antidepressant.

## Methods

### Population

The Swedish bipolar cohort collection (SWEBIC) is a genetic study of bipolar disorder. This cohort has been used for the study of lithium response [18] as well as to identify genetic risk loci for bipolar disorder in international collaborative studies [19]. Participants were mainly recruited via the Swedish Quality Assurance Register for Bipolar Disorder (BipoläR), which benchmarks treatments and outcomes of bipolar management across Sweden [20]. Patients were also enrolled using a validated algorithm [21] applied on the Swedish National Patient Register as well as from the St. Göran Bipolar study, which is a two-center clinical study of bipolar disorder [22]. Individuals could be included at any time during the disease course. The study was approved by the Regional Ethical Review Board in Stockholm, Sweden, and all participants provided written informed consent.

Study participant data were linked to the National Patient Register and the Prescribed Drug Register using Swedish personal identification numbers. The Patient Register contains information on inpatient care since 1968 and specialized outpatient care since 2001, including admission and discharge dates with primary and secondary diagnoses according to the 10th edition of the International Classification of Disease (ICD-10) from 1997 and onwards. The Prescribed Drug Register contains information on all dispensed and purchased drugs in Sweden, including date and size of each dispense. The drugs are coded according to the Anatomical Therapeutic Chemical Classification System (ATC) [23]. The linkage included information from July 2005 up until December 2016, except the Prescribed Drug Register, which included data until December 2019. The present study includes 5019 bipolar patients.

### Genotyping

DNA samples were collected between 2009 and 2013. Those who volunteered after being contacted by mail or phone were interviewed over telephone by trained psychiatric nurses. Blood samples were obtained at the nearest lab and sent to the Karolinska Institutet Biobank [24]. The DNA collection and genotyping procedures in SWEBIC have been described previously [25]. In short, DNA was extracted from whole blood samples stored at the Karolinska Institutet Biobank. Genotyping was conducted in three different waves at the Broad Institute in Boston, US, using Affymetrix 6.0 (Affymetrix, Santa Clara, CA, USA), Illumina OmniExpress (Illumina, San Diego, CA, USA), and Infinium PsychArray-24 v1.2 BeadChip (Illumina, San Diego, CA, USA). Genotypes were imputed to The Haplotype Reference Consortium (HRC) reference panel (version 1.1) on the Sanger imputation server [26].

Phased genotype information on two single nucleotide polymorphisms (SNPs) in *CYP2C19* were included in our analyses: rs4244285-A (minor allele frequency [MAF] = 0.147) and rs12248560-T (MAF = 0.19), which are the most common functional *CYP2C19* polymorphisms in persons of European ancestry [27]. Both SNPs had an imputation info score >0.99. We used the CYP naming convention where CYP2C19*1 is the wild type allele. Specifically, rs4244285-A (CYP2C19*2) is a non-functional allele, and rs12248560-T (CYP2C19*17) is an increase of function allele. We designated *CYP2C19* metabolic phenotypes based on a previously published diplotype scheme: Poor metabolizers (PM, *2/*2), intermediate metabolizers (IM, *2/*1, intermediate+ metabolizers (IM + , *2/*17), extensive metabolizers (EM, also known as normal metabolizers, *1/*1), extensive+ metabolizers (EM+ , *1/*17), and ultra-rapid metabolizers (UM, *17/*17) [27].

In follow-up analyses, we expanded the haplotype analyses to also include the variants rs2860840 (C > T, MAF = 0.39, INFO > 0.99) and rs11188059 (G > A, MAF = 0.17, INFO > 0.89) that can be used to derive the ultra-rapid CYP2C:TG haplotype based on a recent report where the authors studied patients receiving escitalopram [28] The frequencies of the different diplotypes can be seen in Table S2.

### Treatment periods

We used individual series of antidepressant dispensation dates from the Prescribed Drug Register to estimate treatment periods for citalopram, escitalopram, sertraline, amitriptyline, and clomipramine. If the length between dispense dates exceeded 4 months (122 days), the last treatment date was defined as the last date of dispense date plus the number of defined daily doses (DDD) allotted at the last dispense, with a maximum of 90 days added. Four months was chosen between dispense dates because although Swedish drug dispenses is limited to 3 months supply within the drug reimbursement program, a new purchase can be made after two thirds of estimated consumption time of the dispense. The treatment length of a single drug dispense was equal to the amount of DDDs dispensed. The DDDs for the different medications were 20 mg citalopram, 10 mg escitalopram, 50 mg sertraline, 100 mg clomipramine, and 75 mg amitriptyline [29]. As individuals could have started treatment before July 1st 2005, we only included treatments that started after 31st October 2005 (122 days after study start). Since we had data on dispensed medication until 2019 and could thus determine if the individuals were treated at December 31st 2016 (study end).

### Outcomes and statistical methods

To test the impact of *CYP2C19* metabolic phenotypes on four outcomes, we used a categorical variable where extensive (i.e., ‘normal’) metabolizers served as the reference category.

In a first analysis, we used our treatment periods to explore if patients claimed a second dispense after the first dispense to measure if early treatment non-persistence [30] was associated with *CYP2C19* metabolic phenotype. We counted the number of dispenses in each treatment period and the outcome was dichotomized to a refill within 4 months of the initial dispense or not. We excluded individuals who died within 4 months of the first dispense. Given that individuals potentially could have more than one treatment period, and that outcomes from the same individual are potentially correlated, we used generalized estimating equations (GEE) with logistic regression. We used an exchangeable correlation structure, which assumes that the observations from the same individual have the same correlation across different time-points [31]. In a secondary analysis, we tested if the effect of CYP2C19 was dependent on whether patients received more than one antidepressant drug (defined as receiving another medication from antidepressant ATC:N06A within 4 months before or at study start). This variable was added both as a main effect and as an interaction term to the CYP2C19 metabolic phenotype variables. We thereafter tested whether the inclusion of the interaction effects was significant using a Wald test, comparing the nested models with or without the interaction term. In a sensitivity analysis, we allowed for 6 months between the first and second dispense date. Analyses were adjusted for sex, study wave, and age at treatment start. Furthermore, we also analysed the data using the expanded dipolotype scheme from Bråten et al. [28] with the diplotype “CYP2C:CG or TA/ CYP2C:CG or TA” as the reference level.

Second, we constructed a survival analysis of treatment discontinuation during the first year of treatment using the treatment periods. Patients were followed from their first dispense until treatment discontinuation, defined as the last day of treatment. Individuals were censored at the end of the study period (December 31st 2016), death, or after 365 days, whichever came first. Since individuals could have more than one treatment period, We used Cox regression with robust standard errors to correct for non-independence of observations from the same individual using the cluster function in the *survival* package in R [32]. Analyses were adjusted for sex, study wave, and age at treatment start. We performed several sensitivity analyses to test the robustness of our results. We also tested whether an inclusion of an interaction with monothereapy was significant using the same method as described above for early treatment non-persistence. First, we conducted an analysis where we only used the first treatment period for each individual. Second, we allowed 6 months instead of 4 months between dispense dates. Third, we conducted an analysis where we excluded co-medication with the enzyme inducers carbamazepine, phenobarbital, and phenytoin, as well as the enzyme inhibitors omeprazole, esomeprazole, lansoprazole, pantoprazole, fluoxetine, and fluvoxamine [8, 33]. Medication with these drugs were defined as a dispense of the drug within 6 months of treatment start. Fourth, we also analysed the data using the expanded dipolotype scheme from Bråten et al. [28] with the diplotype “CYP2C:CG or TA/ CYP2C:CG or TA” as the reference level.

The third outcome measure was switch to another antidepressant medication within 1 year of treatment initiation. In this analysis, we used another method to establish start of a treatment period in order to make our results comparable with previous studies on *CYP2C19* metabolic phenotypes and antidepressant treatment [10]. We divided the follow-up period July 1st 2006 to December 31st 2016 into monthly segments. Patients were included if they had a drug dispense of either citalopram, escitalopram, sertraline, clomipramine, or amitriptyline. We then excluded subjects prescribed an antidepressant (ATC-group: N06A) during the previous year to ascertain that it was a new treatment period and not an add-on treatment itself. The outcome was a prescription of a new antidepressant of another type within 1 year from treatment start. Individuals were followed from the date of drug dispense until a new antidepressant was prescribed, and were censored at death, study end (December 31st 2016) or after 365 days, whichever came first. Analyses were adjusted for sex, study wave, and age at treatment start. We used Cox regression with robust standard errors to correct for non-independence of observations from the same individual. Furthermore, we also analysed the data using the expanded dipolotype scheme from Bråten et al. [28] with the diplotype “CYP2C:CG or TA/ CYP2C:CG or TA” as the reference level.

In the fourth outcome analysis, we tested the association between *CYP2C19* metabolic phenotypes and manic episodes emerging within 3 months after initiation of antidepressant treatment. In this analysis, we used the treatment periods defined from the second outcome analysis (discontinuation during the first year of treatment). We used hospital admissions with a discharge diagnosis of mania or mixed episode within 3 months [5] of antidepressant treatment start as the outcome (ICD-10 codes F300–302, F308–309, F310–312, and F316). Patients were followed from the first dispense of an antidepressant until hospital admission and were censored at end of follow-up (3 months after treatment initiation), or death, whichever came first. We used Cox regression analysis adjusted for age at treatment start, study wave, and sex. Using robust standard errors to correct for non-independence of observations from the same individual. We hypothesized that the relationship between CYP2C19 function and risk for antidepressant induced mania would be positively related to metabolizing activity as defined by the metabolic phenotype. We therefore also report an analysis where *CYP2C19* metabolic phenotypes was tested as a six-level continuous variable or an inverted ‘activity score’ where UM was 1 and PM was 6 [34]. We furthermore performed an analysis where we adjusted for mood stabilizing medication at treatment start. Medication with mood stabilizing drugs (lithium, valproate, and lamotrigine) was defined as at least two dispenses within the last year and one of these within the last 4 months before the start of the treatment period. We also tested whether an inclusion of an interaction with antidepressant monotherapy was significant using the same method as described above for ‘early persistence’. In this analysis, we used an interaction between the ‘activity score’ and antidepressant monotherapy. We also conducted one analysis where we allowed 6 months instead of 4 months between dispense dates as defined in the discontinuation treatment analysis above. Moreover, we used the expanded dipolotype scheme from Bråten et al. [28] in a final analysis with the diplotype “CYP2C:CG or TA/ CYP2C:CG or TA” as the reference level. As there were a limited number of events in this analysis, we limited the categorical variable to three categories: those that had respectively shown lower and higher plasma concentrations of escitalopram than the reference category, “CYP2C:CG or TA/ CYP2C:CG or TA”, in Bråten et al. [28]. Finally, we used the diplotype scheme ranging from 1 (CYP2C:TG/ CYP2C19*17) to 10 (CYP2C19null/ CYP2C19null) as a continuous variable (Table S2).

## Results

Our sample consisted of 5019 bipolar patients who were genotyped and had registry linkage data available. The characteristics, genotypes, and classification according to metabolic phenotype of the patients are shown in Table 1. Patients were on average 49 years old when patient inclusion began in 2009, 62% of the sample were women, and 44% had a bipolar I disorder diagnosis. The high mean age at inclusion reflects the fact that patients could be included at any time during the course of illness. Between July 2005 and December 2016, 78% of patients had at least one dispense of any antidepressant medication (ATC: N06A). The respective percentages for the drugs metabolized by CYP2C19 were: citalopram 23%, escitalopram 23%, sertraline 27%, amitriptyline 8%, clomipramine 8% (Table S1).

**Table 1:** Characteristics of the sample (*n* = 5019).

Mean age January 1st 2009 (mean, SD)	49.2 (14.8)
Male, *n* (%)	1893 (37.7)
Bipolar subtype *n* (%)	
Type 1	2188 (43.6)
Type 2	1648 (32.8)
UNS	975 (19.4)
Schizoaffective/Cyclothymia	72 (1.4)
Missing information	138 (2.7)
Deaths during follow-up *n* (%)	344 (6.9)
rs4244285, *n* (%)	
A/A	95 (1.9)
G/A	1288 (25.7)
G/G	3636 (72.4)
rs12248560, *n* (%)	
C/C	3274 (65.2)
C/T	1565 (31.2)
T/T	180 (3.6)
*CYP2C19* metabolic phenotype, *n* (%)	
PM	95 (1.9)
IM	992 (19.8)
IM+	296 (5.9)
EM	2187 (43.6)
EM+	1269 (25.3)
UM	180 (3.6)

*PM* poor metabolizer, *IM* intermediate metabolizer, *IM+* intermediate+ metabolizer, *EM* extensive metabolizers, *EM* extensive+ metabolizers, and *UM* ultra-rapid metabolizers.

**Fig. 1:**
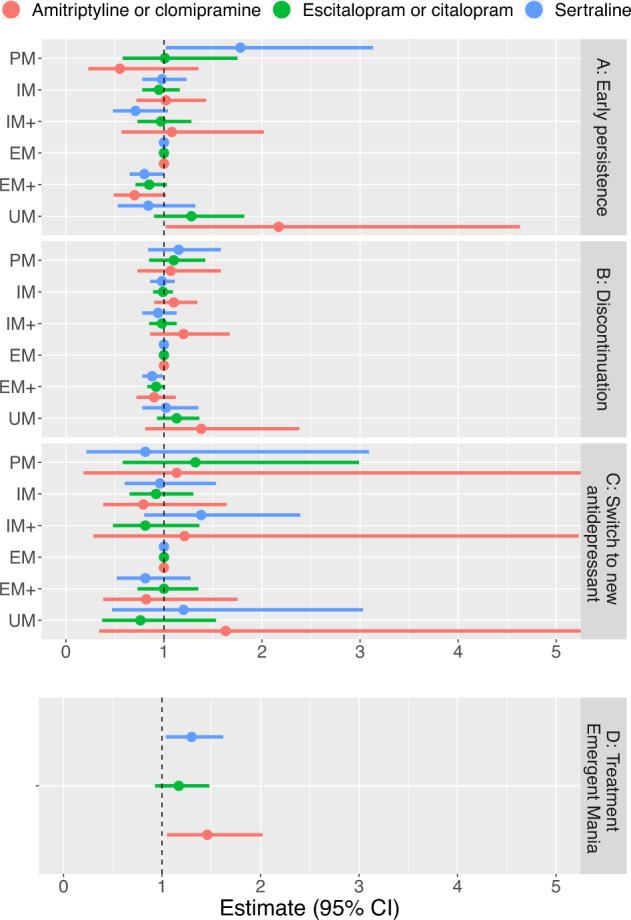
Results from analyses of medication patterns and outcomes and their associations with CYP2C19 metabolic phenotypes: poor metabolizer (PM), intermediate metabolizer (IM), intermediate+ metabolizer (IM+), extensive metabolizers (EM), extensive+ metabolizers (EM+), and ultra-rapid metabolizers (UM). Each medication group is analyzed separately for each outcome but presented together for clarity. *Full estimates are presented in* Tables 2–4 and S12 in the online data supplement. **A** Early treatment non-persistence defined as not collecting medication within 4 months of the first medication dispense. Results are presented as odds ratios and 95% confidence interval (CI). Higher odds ratio indicates higher odds of not collecting a second dispense compared to the Extensive Metabolizer (EM) group. **B** Discontinuation within 1 year of treatment start based on gaps between dispenses of the medication in question. Results presented as Hazard Ratios (HR) and 95% CI where a higher HR is interpreted as having a higher risk of discontinuation compared with the Extensive Metabolizer (EM) group. **C** Treatment switch to another antidepressant within 1 year of treatment start. Results presented as HR with 95% CI where a higher HR indicates a higher risk of treatment switch compared with the Extensive Metabolizer (EM) group. Note that some CI:s extends beyond the limits of the plot window. **D** Antidepressant treatment emergent mania defined as an inpatient diagnosis of mania within 3 months of treatment start. CYP2C19 metabolic phenotype operationalized as a continuous variable going from the UM to the PM metabolic phenotype. Results presented as HR and 95% CI with higher HR indicating an association between less extensive or slower metabolism and a higher risk of treatment emergent mania.

Results on early treatment non-persistence are shown in Fig. 1 and Table 2. We found a higher risk of not claiming a second dispense of sertraline within 4 months in PMs (OR = 1.78, 95%; CI = 1.02–3.13, *p* = 0.043) and lower risk among EM+ :s (OR = 0.8, 95% CI = 0.65–1, *p* = 0.048) as compared with EM:s. Among amitriptyline and clomipramine users, UM:s (OR = 2.17, 95% CI = 1.02–4.63, *p* = 0.044) had a higher risk of not claiming a second dispense compared with EM:s. In a sensitivity analysis using 6 months between dispense dates as cut-off to distinguish different treatment periods, the point estimates were similar but not significant (see Table S3). Furthermore, the inclusion of an interaction term with monotherapy at study start was not significant (see Table S4). Patients not on monotherapy were 23%, 21%, and 41% for escitalopram or citalopram, sertraline, and amitriptyline or clomipramine respectively (Table S4). The analysis using the expanded dipolotype scheme [28] showed a lower risk of not purchasing a second dispense of patient with the TG/*17 and *17/null diplotypes compared to the reference group (CYP2C:CG or TA/ CYP2C:CG or TA) among sertraline users (see Table S5).

**Table 2:** Association between not collecting another dispense within the first 4 months of the initial dispense (early treatment non-persistence) and *CYP2C19* metabolic phenotype. Results presented as odds ration with 95% CI.

	PM	IM	IM+	EM	EM+	UM	*n*	Unique individuals, *n*	Events
Escitalopram or citalopram	1.01 (0.58–1.75)	0.95 (0.78–1.16)	0.97 (0.73–1.28)	1	0.85 (0.71–1.03)	1.28 (0.9–1.82)	5218	1724	1484
Sertraline	1.78 (1.02–3.13)	0.98 (0.78–1.23)	0.71 (0.48–1.04)	1	0.8 (0.65–1)	0.84 (0.53–1.32)	3336	1168	1012
Amitriptyline or clomipramine	0.55 (0.23–1.35)	1.02 (0.72–1.43)	1.08 (0.57–2.02)	1	0.7 (0.49–1.02)	2.17 (1.02–4.63)	1406	610	422

All analyses are adjusted for age at start of medication period, study wave, and sex.

*PM* poor metabolizer, *IM* intermediate metabolizer, *IM+* intermediate+ metabolizer, *EM* extensive metabolizers, *EM* extensive+ metabolizers, and *UM* ultra-rapid metabolizers.

Results on treatment discontinuation within the first 12 months are shown in Fig. 1 and Table 3. We found a lower risk of sertraline treatment discontinuation among EM+ compared with EM (HR 0.88, 95% CI: 0.78–0.99, *p* = 0.034). This did not persist in sensitivity analyses (see Tables S7–S9). We also report median times until discontinuation for each metabolic phenotype these are shown in Table S4 (these are based on the analyses reported in Table 3). The median treatment durations for amitriptyline and clomipramine antidepressants stand out in these analyses, 37.8 days compared to most other treatment times that are closer to 180 days. We further noted that there indeed was a higher risk for early discontinuation in the first 2 months of the follow-up and there seemed to be a deviation of proportional hazards assumption in the main model that used the full 12 months of follow-up. We, therefore, performed an additional analysis where we only used follow-up times for the first 60 days. These results show a higher risk for discontinuation for UM compared to EM metabolizers: HR: 2.54 (95% CI: 1.38–4.67) among patients treated with amitriptyline or clomipramine. Furthermore, the inclusion of an interaction term with monotherapy at study start was not significant (see Table S10). The analysis using the expanded dipolotype scheme [28] showed a lower risk of discontinuation of patient with the TG/*17 and TG/TG diplotypes compared to the reference group (CYP2C:CG or TA/ CYP2C:CG or TA) among sertraline users (see Table S11).

**Table 3:** Association between *CYP2C19* metabolic phentoype and treatment discontinuation within 1 year of initiation.

	PM	IM	IM +	EM	EM +	UM	*n*	Events	Unique individuals, *n*	Median treatment times in days
Escitalopram or citalopram	1.1 (0.85–1.42)	0.99 (0.89–1.09)	0.98 (0.85–1.13)	1	0.92 (0.83–1.01)	1.13 (0.93–1.36)	5487	3965	1846	189
Sertraline	1.15 (0.84–1.58)	0.98 (0.86–1.11)	0.94 (0.78–1.13)	1	0.88 (0.78–0.99)	1.02 (0.78–1.35)	3484	2422	1253	191
Amitriptyline or clomipramine	1.07 (0.73–1.58)	1.1 (0.9–1.34)	1.2 (0.86–1.67)	1	0.9 (0.72–1.12)	1.38 (0.81–2.38)	1524	1035	668	176

All analyses are adjusted age at start of medication period, study wave, and sex.

Results are presented as hazard ratios (95% CI).

*PM* poor metabolizer, *IM* intermediate metabolizer, *IM+* intermediate+ metabolizer, *EM* extensive metabolizers, *EM* extensive+ metabolizers, and *UM* ultra-rapid metabolizers.

The likelihood of switching to another antidepressant are presented in Fig. 1 and Table S12. The number of patients in each analysis are shown in Supplementary Figs. S1–3. These analyses did not reveal any increased likelihood of switching to new antidepressants for different metabolic phenotypes when compared to EM:s. The analysis using the expanded dipolotype scheme [28] did not reveal any significant associations (Table S13).

For analyses of treatment emergent manic episodes (Fig. 1 and Table 4), we collapsed the patients into three groups (PM/IM/IM+ , EM, and EM+ /UM) due to the limited number of events. We found no significant association between *CYP2C19* metabolic phenotypes and treatment emergent manic episodes. However, when we used *CYP2C19* metabolic phenotypes as a continuous variable (from ultra-rapid to poor metabolizers in six steps), we found that slower metabolism was associated with a higher risk of mania during the first 3 months of sertraline treatment (HR 1.3, 95% CI 1.04–1.62, *p* = 0.020) and amitriptyline or clomipramine treatment (HR 1.46, 95% CI: 1.05–2.02, *p* = 0.024). Furthermore, the inclusion of an interaction term with monotherapy at study start was not significant (see Table S15). Adjusting for mood stabilizing treatment did not change the results (Table S16). A sensitivity analysis using 6 months instead of 4 months as allowable time between dispense dates (Table S14) showed similar results. We did not adjust for study wave in analyses of sertraline and the analysis of amitriptyline and clomipramine in sensitivity analyses because some study waves did not have any events. The analysis using the expanded dipolotype scheme [28] did not reveal any significant associations when we used a three-level categorical variable but did show a significant effect when used a continuous variable with slower metabolism being associated with higher risk of treatment emergent mania for amitriptyline or clomipramine but not for sertraline (Table S17).

**Table 4:** Association between CYP2C19 metabolic phenotype and risk of treatment emergent mania within the first 3 months of starting antidepressant treatment.

	PM/IM/IM+	EM	EM + /UM	HR for trend	*P*-value for trend	*n*	Events	Unique individuals, *n*
Escitalopram or citalopram	1.59 (0.85–2.99)	1	1.05 (0.51–2.14)	1.17 (0.93–1.48)	0.175	5487	54	1846
Sertraline^a^	1.48 (0.76–2.87)	1	0.41 (0.16–1.03)	1.3 (1.04–1.62)	0.020	3484	50	1253
Amitriptyline or clomipramine	2.76 (0.82–9.27)	1	0.67 (0.12–3.73)	1.46 (1.05–2.02)	0.024	1524	18	668

All analyses are adjusted for age at start of medication period, study wave, and sex.

*PM* poor metabolizer, *IM* intermediate metabolizer, *IM+* intermediate+ metabolizer, *EM* extensive metabolizers, *EM* extensive+ metabolizers, and *UM* ultra-rapid metabolizers.

Results presented as hazard ratios (95% CI).

^a^Not adjusted for study wave due to lack of outcomes in some waves.

## Discussion

In this study of more than 5000 patients with bipolar disorder, we found an association between *CYP2C19* metabolic phenotypes and a manic episode within 3 months of starting treatment with either sertraline or the tricyclic antidepressants amitriptyline and clomipramine. This suggests that individual differences in drug metabolism influence the risk for treatment emergent mania and point toward a dose response association. Although differences in metabolic phenotype influenced early treatment non-persistence of sertraline, clomipramine, and amitriptyline, these results did not hold in sensitivity analyses. All things considered, our results show little impact of *CYP2C19* metabolic phenotypes on early treatment persistence, treatment discontinuation, or switch to another antidepressant in bipolar disorder patients taking sertraline, citalopram, escitalopram, amitriptyline, and clomipramine.

This is the first large scale study on the importance of *CYP2C19* metabolic phenotypes for antidepressant treatment patterns and adverse events in bipolar disorder patients. But there are previous large studies on other patient groups using citalopram or escitalopram [10, 27, 34]. A meta-analysis of three studies in major depressive disorder found a better antidepressant effect and higher risk of early side effects in poor metabolizers compared with extensive metabolizers [27]. Notably, this meta-analysis had access to detailed short term follow-up data, which was not available in our register-based data. Yet their results are not necessarily at odds with ours as they also did not find *CYP2C19* metabolic phenotypes to be associated with drop-out rates. Jukić and colleagues found a higher rate of treatment failure of escitalopram in ultra-rapid and poor metabolizer compared with extensive metabolizers [10], a finding that we could not replicate in our study. One possible explanation is that we defined the outcome differently: Whereas our endpoint was a prescription of a new antidepressant within a year of the first dispense, Jukić and colleagues used the last therapeutic drug monitoring (TDM) analysis as starting point, which can occur at any timepoint during treatment. Furthermore, the study of Jukić and colleagues did not specify the diagnosis of included patients whereas we only included patients with bipolar disorder.

With respect to sertraline, we found that PM had a lower chance of early persistence, i.e., claiming a second dispense. However, this association was not significant in sensitivity analyses. We also found a lower risk of treatment discontinuation in patients with the *CYP2C19* EM+ metabolic phenotype, an effect that persisted in some sensitivity analyses. This observation could indicate a lower rate of side effects that has not been described in previous studies. Although higher plasma concentration of sertraline has been reported in poor CYP2C19 metabolizers [6, 8, 9], there is scant evidence to suggest that *CYP2C19* metabolic phenotypes affect treatment outcomes or adverse events [6, 35].

The CPIC guideline recommends that tricyclics should be avoided in CYP2C19 ultra-rapid metabolizers [7]. CYP2C19 metabolizes amitriptyline into the active metabolite nortriptyline, and clomipramine into the active metabolite desmethylclomipramine. The drug/metabolite ratio has been shown to differ based on *CYP2C19* genotype [13, 36]. This might factor in on response and side effect profile due different effects of secondary amines such as nortryptiline [7]. Here we present the first empirical evidence that this recommendation might extend to bipolar disorder. The rate of early persistence was lower among ultra-rapid metabolizers using clomipramine or amitriptyline. Although the association did not remain in sensitivity analyses, it suggests that *CYP2C19* metabolic phenotypes might be of importance for early treatment discontinuation.

A biomarker that can predict manic switch when using antidepressants in bipolar disorder would be valuable. We found slower drug metabolism via *CYP2C19* to be associated with a higher rate of manic episodes within 3 months after starting treatment with sertraline and with amitriptyline or clomipramine. It stands to reason that a slower metabolism with ensuing higher serum concentrations might be a risk factor for treatment emergent mania. By extension, this indicates a likely dose-response relationship between antidepressant dose and risk for treatment emergent mania. Interestingly, higher risk of treatment induced mania has previously been linked to poor CYP2D6 metabolizers, but data are limited [37, 38]. It should be noted that the although the results did replicate in sensitivity analyses, the event rate was low, the p-value close to 0.05, and was one among several other tested hypotheses. The finding should therefore be interpreted with caution. Future replication studies of adequate power might attempt to genotype both *CYP2D6* and *CYP2C19*. It should be noted that these studies need be large given the low number events found in our study that amounted to 50 manic inpatient events during the first 3 months of 3484 sertraline treatments periods.

We used CYP2C19*2 and CYP2C19*17 which are the most common variants of CYP2C19. Recently it was however reported that the CYP2C:TG haplotype (rs2860840C > T and rs11188059G > A) was associated with faster metabolism of escitalopram [28]. We performed additional analyses for all endpoints using the diplotype scheme given by Bråten et al. This changed very little in the overall interpretation of our analysis.

There are inevitable limitations even with a large genotyped sample of bipolar disorder patients with full coverage of purchased drug treatments. First, the adherence to dispensed drugs is unknown in register data. Second, the length of the treatment exposure is calculated by a series of drug dispenses and the number of dispensed DDDs at the last drug dispense. Although this provides a rough estimate of treatment exposure, potential stockpiling and dosage changes might affect actual treatment periods. However, we also tested our results in sensitivity analyses allowing for 6 months instead of 4 months between dispensed medications. Third, amitriptyline is an antidepressant but may also be used to treat neuropathic pain at lower doses than used to treat depression. As we do not have information on prescribed daily doses or indication for treatment, we cannot distinguish between specific indications for treatment although depression is the most likely indication for amitriptyline in a bipolar disorder cohort. The high prevalence of not being on antidepressant monotherapy might be due to tricyclic antidepressants being used for other indications. Fourth, combining amitriptyline and clomipramine into one group might be questioned as the recommendations regarding clomipramine from CPIC are based largely on extrapolating data on amitriptyline [7]. We combined them because of the low prevalence of poor and ultra-rapid metabolizers and because the use of these drugs is relatively uncommon. Fifth, we limited this study to two *CYP2C19* polymorphisms because information on rare *CYP2C19* polymorphisms is not readily available from genome-wide data. Including additional polymorphisms would have given a more complete categorization of metabolic phenotypes [7, 10]. Sixth, we used the outcome treatment discontinuation based on the assumption that it was due to adverse events or lack of efficacy. But there are other reasons to discontinue an antidepressant, including successful treatment, and we were unable to distinguish between the reasons for discontinuation in our analyses. To address this, we first limited our follow-up time to the first 12 months of treatment. We also analysed early treatment persistence and switch to another antidepressant which should be indicative of early side effects, lack of proper evaluation, or treatment failure. Seventh, in a recent analysis of the effect of *CYP2C19* metabolic phenotypes on escitalopram/citalopram treatment, the authors argued that multiple testing correction should solely be based on the number independent outcomes and not take into account the six different groups of *CYP2C19* metabolic phenotypes as these were “deemed consolidated classification with precise functional and biological meaning” [27]. We have instead opted to include a number of sensitivity analyses to test the robustness of our findings as they are based on a number of assumptions regarding dispense records. The results should, however, be viewed in context of multiple testing and findings close to the 0.05 limit that are not supported by sensitivity analyses should certainly be interpreted with caution [39]. Eight, although we have a very large sample, the UM and PM phenotypes are still rare in this cohort reducing our power to find smaller effects especially for rarer events such as inpatient care for mania. For instance, for 80% power to find a 1.2 HR between two groups when one of the groups is 3.6% of the sample and the overall event rate is 50% would require a sample of 13,580. For an HR of 1.5 and otherwise same specifications the sample size required is 2746. We can leverage our longitudinal follow-up by using several treatment periods per individual yet these are non-independent observation and some medications are not prevalently used thus limiting our study to finding mid- to large sized effects.

In conclusion, this is the first large scale study of the impact of *CYP2C19* metabolic phenotypes on antidepressant treatment patterns and adverse events in bipolar disorder patients. We report largely negative results on antidepressant discontinuation and treatment switch. However, our results indicate a possible association between slower CYP2C19 metabolism and antidepressant treatment emergent mania in patients treated with sertraline and amitriptyline. This finding warrants future explorations.

## Supplementary information

online data supplement

## Data Availability

All materials and datasets generated and/or analyzed during the current study are available from the corresponding author on reasonable request.
