# Supplementary material for: Effect of CYP2C19 polymorphisms on antidepressant prescription patterns and treatment emergent mania in bipolar disorder
Source: Pharmacogenomics J. 2022 Nov 4;23(1):28–35. doi: 10.1038/s41397-022-00294-4 (PMC9925376; doi:10.1038/s41397-022-00294-4)
Supplement: Supplementary file 1 — online data supplement [file 41397_2022_294_MOESM1_ESM.docx]

| Table s1. Any dispensed antidepressant medication during follow-up (1^st^ July 2005 until 31^st^ December 2016) stratified by *CYP2C19* metabolic phenotype | | | | | | | |
| --- | --- | --- | --- | --- | --- | --- | --- |
|  | PM | IM | IM+ | EM | EM+ | UM | Total |
| n | 95 | 992 | 296 | 2187 | 1269 | 180 | 5019 |
| Citalopram, N (%) | 25 (26.3) | 224 (22.6) | 73 (24.7) | 518 (23.7) | 280 (22.1) | 40 (22.2) | 1160 (23.1) |
| Escitalopram, N (%) | 20 (21.1) | 229 (23.1) | 75 (25.3) | 503 (23.0) | 283 (22.3) | 44 (24.4) | 1154 (23.0) |
| Sertraline, N (%) | 21 (22.1) | 259 (26.1) | 89 (30.1) | 612 (28.0) | 328 (25.8) | 54 (30.0) | 1363 (27.2) |
| Amitriptyline, N (%) | 8 (8.4) | 95 ( 9.6) | 18 (6.1) | 185 (8.5) | 93 (7.3) | 18 (10.0) | 417 (8.3) |
| Clomipramine, N (%) | 9 (9.5) | 76 (7.7) | 20 (6.8) | 167 (7.6) | 96 (7.6) | 10 (5.6) | 378 (7.5) |
| Any antidepressant*, N (%) | 70 (73.7) | 769 (77.5) | 242 (81.8) | 1734 (79.3) | 975 (76.8) | 147 (81.7) | 3937 (78.4) |
| Any antidepressant that is not a CYP2C19 substrate^†^, N (%) | 47 (49.5) | 610 (61.5) | 185 (62.5) | 1395 (63.8) | 773 (60.9) | 120 (66.7) | 3130 (62.4) |
| * any medication in the ATC N06A subgroup  ^†^ any medication from ATC N06A subgroup that is not Citalopram, Escitalopram, Sertraline, Amitriptyline, or Clomipramine  Abbreviations: poor metabolizer (PM), intermediate metabolizer (IM), intermediate+ metabolizer (IM+), extensive metabolizers (EM), extensive+ metabolizers (EM), and ultra-rapid metabolizers (UM) | | | | | | | |

| Table s2. Frequency of individuals with different diplotypes in SWEBIC, adapted from Bråten et al (2021). | |
| --- | --- |
| Diplotype | Overall (N=5016*) |
|  |  |
| 1. CYP2C:TG/ CYP2C19*17 | 424 (8.5%) |
| 2. CYP2C:TG/ CYP2C:TG | 247 (4.9%) |
| 4. CYP2C19**17/* CYP2C19*17 | 178 (3.5%) |
| 5. CYP2C:TG / CYP2C:CG or TA | 969 (19.3%) |
| 6. CYP2C19*17 / CYP2C:CG or TA | 844 (16.8%) |
| 7.EM (CYP2C:CG or TA/ CYP2C:CG or TA) | 971 (19.4%) |
| 8. CYP2C:TG/ CYP2C19null | 342 (6.8%) |
| 9. CYP2C19*17/ CYP2C19null | 296 (5.9%) |
| 10. CYP2C19null/ CYP2C:CG or TA | 650 (13.0%) |
| 11. CYP2C19null/ CYP2C19null | 95 (1.9%) |
|  |  |
| *We did not include three individuals with diplotypes that were not described in Bråten et al (2021).  This includes CYP2C:TG_CYP2C19*17/ CYP2C19*17 (n=2) and TG_ CYP2C19*17/CYP2C:CG or TA (n=1) | |

| Table s3. Association between not purchasing another dispense within the first six months of the initial dispense and *CYP2C19* metabolic phentoype using six months between medication dispenses as a cut-off for continuous treatment periods. Results are presented as odds ratios with 95% CI. | | | | | | | | | |
| --- | --- | --- | --- | --- | --- | --- | --- | --- | --- |
|  | PM | IM | IM+ | EM | EM+ | UM | n | Unique individuals, n | Events |
| Escitalopram or citalopram | 0.79 (0.38-1.65) | 0.85 (0.67-1.09) | 0.82 (0.55-1.22) | 1 | 0.86 (0.68-1.09) | 1.39 (0.91-2.11) | 3066 | 1728 | 689 |
| Sertraline | 1.38 (0.58-3.29) | 1.02 (0.75-1.41) | 0.88 (0.49-1.56) | 1 | 0.91 (0.68-1.21) | 1.4 (0.79-2.51) | 1829 | 1087 | 431 |
| Amitriptyline or clomipramine | 0.35 (0.14-0.88) | 0.97 (0.66-1.44) | 1.04 (0.5-2.14) | 1 | 0.54 (0.35-0.84) | 2.13 (0.9-5.03) | 853 | 550 | 249 |
| All analyses are adjusted for age at start of medication period, study wave, and sex.  Abbreviations: poor metabolizer (PM), intermediate metabolizer (IM), intermediate+ metabolizer (IM+), extensive metabolizers (EM), extensive+ metabolizers (EM), and ultra-rapid metabolizers (UM) | | | | | | | | | |

| Table s4. Output showing p-value for interaction term between not being on monotherapy of study drugs and CYP2C19 metabolic phenotype and early treatment persistence. | | |
| --- | --- | --- |
|  | Not on monotherapy* n(%) | P-value of interaction term |
| Escitalopram or citalopram | 1174 (22.5) | 0.78 |
| Sertraline | 662 (19.8) | 0.59 |
| Amitriptyline or clomipramine | 547 (38.9) | 0.81 |
| *Also treated with another antidepressant (ATC N06A) at treatment start | | |

| Table s5. Association between not purchasing another dispense within the first four months of the initial dispense and *CYP2C* diplotypes using 4 months between medication dispenses as a cut-off for continuous treatment periods. Results are presented as odds ratios with 95% CI. | | | | | | | | | | | | | |
| --- | --- | --- | --- | --- | --- | --- | --- | --- | --- | --- | --- | --- | --- |
|  | CYP2C19null/ CYP2C19null | CYP2C19null/ CYP2C:CG or TA | CYP2C19*17/ CYP2C19null | CYP2C:TG/ CYP2C19null | Ref* | CYP2C19*17 / CYP2C:CG or TA | CYP2C:TG / CYP2C:CG or TA | CYP2C19**17/* CYP2C19*17 | CYP2C:TG/ CYP2C:TG | CYP2C:TG/ CYP2C19*17 | n | Unique id. | events |
| Escitalopram or citalopram | 1.06 (0.6-1.86) | 0.99 (0.76-1.28) | 1.02 (0.75-1.38) | 1.03 (0.74-1.43) | 1 | 0.94 (0.73-1.21) | 1.13 (0.89-1.43) | 1.35 (0.93-1.96) | 0.94 (0.63-1.4) | 0.82 (0.61-1.1) | 5218 | 1724 | 1484 |
| Sertraline | 1.63 (0.91-2.92) | 0.98 (0.72-1.32) | 0.65 (0.43-0.99) | 0.76 (0.52-1.13) | 1 | 0.77 (0.57-1.03) | 0.9 (0.69-1.19) | 0.77 (0.48-1.24) | 0.71 (0.46-1.08) | 0.68 (0.48-0.97) | 3336 | 1168 | 1012 |
| Amitriptyline or clomipramine | 0.55 (0.22-1.4) | 1.01 (0.62-1.66) | 1.07 (0.54-2.13) | 1.01 (0.62-1.67) | 1 | 0.7 (0.42-1.14) | 1.08 (0.69-1.69) | 1.94 (0.86-4.37) | 0.66 (0.33-1.32) | 0.73 (0.39-1.35) | 1403 | 608 | 420 |
| * CYP2C:CG or TA/ CYP2C:CG or TA  All analyses are adjusted for age at start of medication period, study wave, and sex. | | | | | | | | | | | | | |

| Table s6. Median treatment times in days for different *CYP2C19* metabolic phenotypes in the analysis presented in table 3. | | | |
| --- | --- | --- | --- |
|  | Escitalopram or Citalopram | Sertraline | Amitriptyline or Clomipramine |
| PM | 174 | 170 | 179.0 |
| IM | 184 | 186 | 165.7 |
| IM+ | 191 | 194 | 190.0 |
| EM | 186 | 185 | 175.0 |
| EM+ | 205 | 212 | 195.0 |
| UM | 148 | 186 | 37.8 |

| Table s7. Association between *CYP2C19* metabolic phentoype and treatment discontinuation during the first 12 months after starting antidepressant treatment excluding co-medication that could interfere with metabolism | | | | | | | | | |
| --- | --- | --- | --- | --- | --- | --- | --- | --- | --- |
|  | PM | IM | IM+ | EM | EM+ | UM | n | Events | Unique individuals |
| Escitalopram or citalopram | 1.09 (0.83-1.44) | 1.01 (0.91-1.12) | 0.96 (0.83-1.13) | 1 | 0.92 (0.83-1.02) | 1.1 (0.89-1.36) | 4628 | 3347 | 1655 |
| Sertraline | 1.17 (0.83-1.66) | 0.98 (0.85-1.12) | 1.04 (0.86-1.25) | 1 | 0.9 (0.79-1.02) | 1.06 (0.82-1.37) | 2999 | 2123 | 1099 |
| Amitriptyline or clomipramine | 1.55 (1.03-2.36) | 1.26 (1.02-1.56) | 1.12 (0.79-1.59) | 1 | 0.92 (0.7-1.2) | 1.41 (0.77-2.56) | 1159 | 781 | 528 |
| All analyses are adjusted for number of previous medication periods, age at start of medication period, study wave, and sex.  Abbreviations: poor metabolizer (PM), intermediate metabolizer (IM), intermediate+ metabolizer (IM+), extensive metabolizers (EM), extensive+ metabolizers (EM), and ultra-rapid metabolizers (UM)  * carbamazepine, phenobarbital, phenytoin, omeprazole, esomeprazole, lansoprazole, pantoprazole, fluoxetine, and fluvoxamine | | | | | | | | | |

| Table s8. Association between *CYP2C19* metabolic phentoype and discontinuation during the first 12 months after starting antidepressant treatment when using 6 months days as cut-off between dispense dates. Results presented as hazard ratios (95% CI). | | | | | | | | | | |
| --- | --- | --- | --- | --- | --- | --- | --- | --- | --- | --- |
|  | PM | IM | IM+ | EM | EM+ | UM | n | Events | Unique individuals, n | Median treatment times in days |
| Escitalopram or citalopram | 0.92 (0.62-1.38) | 0.9 (0.78-1.04) | 0.78 (0.61-0.98) | 1 | 0.83 (0.72-0.96) | 1.09 (0.82-1.46) | 3066 | 1666 | 1728 | 284 |
| Sertraline | 0.79 (0.46-1.37) | 1.05 (0.88-1.24) | 1.03 (0.78-1.35) | 1 | 0.98 (0.84-1.15) | 1.51 (1.08-2.11) | 1960 | 1101 | 1173 | 275 |
| Amitriptyline or clomipramine | 0.84 (0.51-1.38) | 1.03 (0.83-1.29) | 1.02 (0.69-1.5) | 1 | 0.76 (0.59-0.98) | 1.47 (0.82-2.65) | 951 | 590 | 608 | 191 |
| All analyses are adjusted for age at start of treatment period, study wave, and sex.  Abbreviations: poor metabolizer (PM), intermediate metabolizer (IM), intermediate+ metabolizer (IM+), extensive metabolizers (EM), extensive+ metabolizers (EM), and ultra-rapid metabolizers (UM) | | | | | | | | | | |

| Table s9. Association between *CYP2C19* metabolic phentoype and discontinuation during the first 12 months of treatment using only the first treatment period | | | | | | | | |
| --- | --- | --- | --- | --- | --- | --- | --- | --- |
|  | PM | IM | IM+ | EM | EM+ | UM | n | Events |
| Escitalopram or citalopram | 1.19 (0.81-1.74) | 1.02 (0.88-1.18) | 0.89 (0.71-1.12) | 1 | 0.88 (0.76-1.01) | 1.04 (0.77-1.39) | 1846 | 1306 |
| Sertraline | 0.84 (0.49-1.47) | 0.85 (0.71-1.02) | 0.94 (0.71-1.23) | 1 | 0.85 (0.72-1.01) | 1.16 (0.83-1.62) | 1253 | 894 |
| Amitriptyline or clomipramine | 1.02 (0.52-2) | 1.11 (0.88-1.39) | 1.06 (0.69-1.63) | 1 | 0.75 (0.59-0.95) | 1.16 (0.7-1.94) | 668 | 459 |
| All analyses are adjusted for age at start of medication period, study wave, and sex.  Abbreviations: poor metabolizer (PM), intermediate metabolizer (IM), intermediate+ metabolizer (IM+), extensive metabolizers (EM), extensive+ metabolizers (EM), and ultra-rapid metabolizers (UM) | | | | | | | | |

| Table s10. Output showing p-value for interaction term between not being on monotherapy of study drugs and *CYP2C19* metabolic phenotype and treatment discontinuation during the first 12 months of treatment | | |
| --- | --- | --- |
|  | Not on monotherapy* n(%) | P-value of interaction term |
| Escitalopram or citalopram | 1285 (23.4) | 0.17 |
| Sertraline | 742 (21.3) | 0.54 |
| Amitriptyline or clomipramine | 617 (40.5) | 0.12 |
| *Also treated with another antidepressant (ATC N06A) at treatment start | | |

| Table s11. Association between *CYP2C* diplotypes and treatment discontinuation during the first 12 months after starting antidepressant treatment | | | | | | | | | | | | | |
| --- | --- | --- | --- | --- | --- | --- | --- | --- | --- | --- | --- | --- | --- |
|  | CYP2C19null/ CYP2C19null | CYP2C19null/ CYP2C:CG or TA | CYP2C19*17/ CYP2C19null | CYP2C:TG/ CYP2C19null | **Ref*** | CYP2C19*17 / CYP2C:CG or TA | CYP2C:TG / CYP2C:CG or TA | CYP2C19**17/* CYP2C19*17 | CYP2C:TG/ CYP2C:TG | CYP2C:TG/ CYP2C19*17 | n | Events | Unique individuals, |
| Escitalopram or citalopram | 1.11 (0.85-1.45) | 1.02 (0.89-1.16) | 0.99 (0.85-1.16) | 0.96 (0.82-1.13) | 1 | 0.92 (0.81-1.04) | 1.05 (0.93-1.19) | 1.14 (0.93-1.4) | 0.9 (0.75-1.07) | 0.94 (0.81-1.09) | 5487 | 3965 | 1846 |
| Sertraline | 1.09 (0.78-1.53) | 0.99 (0.83-1.18) | 0.89 (0.73-1.1) | 0.85 (0.68-1.05) | 1 | 0.86 (0.73-1.02) | 0.97 (0.82-1.14) | 0.98 (0.73-1.31) | 0.78 (0.61-0.98) | 0.8 (0.66-0.98) | 3484 | 2422 | 1253 |
| Amitriptyline or clomipramine | 1.06 (0.7-1.59) | 1.06 (0.81-1.39) | 1.18 (0.82-1.7) | 1.13 (0.84-1.52) | 1 | 0.86 (0.66-1.11) | 1.01 (0.78-1.3) | 1.27 (0.73-2.21) | 0.87 (0.59-1.27) | 0.95 (0.63-1.43) | 1521 | 1032 | 668 |
| All analyses are adjusted for age at start of medication period, study wave, and sex.  * CYP2C:CG or TA/ CYP2C:CG or TA | | | | | | | | | | | | | |

| Table s12. Association between *CYP2C19* metabolic phenotype and treatment switch to another antidepressant within 12 months after starting antidepressant treatment. Results are presented as hazard ratios (95% CI). | | | | | | | | | |
| --- | --- | --- | --- | --- | --- | --- | --- | --- | --- |
|  | PM | IM | IM+ | EM | EM+ | UM | n | Unique individuals | Events |
| Escitalopram or citalopram | 1.32 (0.58-2.99) | 0.92 (0.65-1.3) | 0.81 (0.48-1.36) | 1 | 1 (0.73-1.35) | 0.76 (0.37-1.53) | 1039 | 878 | 276 |
| Sertraline | 0.81 (0.21-3.09) | 0.96 (0.6-1.53) | 1.38 (0.8-2.39) | 1 | 0.81 (0.52-1.27) | 1.2 (0.47-3.03) | 623 | 528 | 143 |
| Amitriptyline or clomipramine | 1.13 (0.18-7.08) | 0.79 (0.38-1.64) | 1.21 (0.28-5.23) | 1 | 0.82 (0.38-1.75) | 1.63 (0.34-7.95) | 188 | 172 | 53 |

All analyses are adjusted for age at start of medication period, study wave, and sex.

Abbreviations: poor metabolizer (PM), intermediate metabolizer (IM), intermediate+ metabolizer (IM+), extensive metabolizers (EM), extensive+ metabolizers (EM), and ultra-rapid metabolizers (UM)

| Table s13. Output showing association with CYP2C dipoltypes and treatment switch to another antidepressant within 12 months after starting antidepressant treatment. Results are presented as hazard ratios (95% CI). | | | | | | | | | | | | | |
| --- | --- | --- | --- | --- | --- | --- | --- | --- | --- | --- | --- | --- | --- |
|  | CYP2C19null/ CYP2C19null | CYP2C19null/ CYP2C:CG or TA | CYP2C19*17/ CYP2C19null | CYP2C:TG/ CYP2C19null | Ref***** | CYP2C19*17 / CYP2C:CG or TA | CYP2C:TG / CYP2C:CG or TA | CYP2C19**17/* CYP2C19*17 | CYP2C:TG/ CYP2C:TG | CYP2C:TG/ CYP2C19*17 | n | Unique individuals | Events |
| Escitalopram or citalopram | 1.33 (0.57-3.1) | 1.11 (0.72-1.71) | 0.82 (0.47-1.43) | 0.61 (0.31-1.2) | 1 | 1.01 (0.67-1.53) | 1.05 (0.72-1.54) | 0.76 (0.37-1.59) | 0.89 (0.48-1.66) | 1 (0.61-1.66) | 1039 | 878 | 276 |
| Sertraline | 0.72 (0.18-2.83) | 1.05 (0.58-1.9) | 1.24 (0.67-2.29) | 0.58 (0.25-1.33) | 1 | 0.71 (0.4-1.26) | 0.75 (0.42-1.34) | 1.07 (0.41-2.82) | 1.01 (0.47-2.17) | 0.77 (0.35-1.69) | 623 | 528 | 143 |
| Amitriptyline or clomipramine | 1.44 (0.21-9.83) | 1.03 (0.36-2.92) | 1.55 (0.33-7.38) | 0.97 (0.25-3.79) | 1 | 0.99 (0.34-2.86) | 1.25 (0.47-3.29) | 1.23 (0.11-13.91) | 2.15 (0.67-6.86) | 1.14 (0.32-4.04) | 187 | 172 | 52 |
| All analyses are adjusted for age at start of medication period, study wave, and sex.  * CYP2C:CG or TA/ CYP2C:CG or TA | | | | | | | | | | | | | |

| Table s14. Association between CYP2C19 metabolic phentoype and risk of mania within 3 months of treatment initiation using six months between dispenses to define treatment periods | | | | | | | | |
| --- | --- | --- | --- | --- | --- | --- | --- | --- |
|  | PM/IM/IM+ | EM | UM/EM+ | HR for trend | P-value for trend | n | Events | Unique individuals, n |
| Escitalopram or citalpram | 1.12 (0.51-2.43) | 1 | 0.93 (0.41-2.1) | 1.02 (0.8-1.3) | 0.898 | 3066 | 35 | 1728 |
| Sertraline* | 1.68 (0.8-3.56) | 1 | 0.46 (0.15-1.41) | 1.3 (1.01-1.68) | 0.043 | 1960 | 31 | 1173 |
| Amitriptyline or clomipramine* | 3.25 (1.02-10.37) | 1 | 0.4 (0.05-3.61) | 1.69 (1.18-2.43) | 0.004 | 951 | 14 | 608 |
| All analyses are adjusted for age at start of medication period, study wave, and sex.  Abbreviations: poor metabolizer (PM), intermediate metabolizer (IM), intermediate+ metabolizer (IM+), extensive metabolizers (EM), extensive+ metabolizers (EM), and ultra-rapid metabolizers (UM)  *Not adjusted for study wave due to lack of outcomes in some waves. | | | | | | | | |

| Table s15. Output showing p-value for interaction term between not being on monotherapy of study drugs and CYP2C19 metabolic phenotype and treatment emergent mania 3 months after treatment initiation. | | |
| --- | --- | --- |
|  | Not on monotherapy* n(%) | P-value of interaction term |
| Escitalopram or citalopram | 1285 (23.4) | 0.86 |
| Sertraline | 742 (21.3) | 0.06 |
| Amitriptyline or clomipramine | 617 (40.5) | 0.99 |
| *Also treated with another antidepressant (ATC N06A) at treatment start | | |

| Table s16. Association between CYP2C19 metabolic phenotype and risk of treatment emergent mania within the first 3 months of starting antidepressant treatment. Results presented as hazard ratios (95% CI), adjusted for mood stabilizing treatment (lithium, valproate, or lamotrigine) | | | | | | | | |
| --- | --- | --- | --- | --- | --- | --- | --- | --- |
|  | PM/IM/IM+ | EM | EM+/UM | HR for trend | P-value for trend | n | Events | Unique individuals, n |
| Escitalopram or citalopram | 1.59 (0.84-2.99) | 1 | 1.06 (0.52-2.17) | 1.17 (0.93-1.48) | 0.181 | 5487 | 54 | 1846 |
| Sertraline* | 1.47 (0.76-2.87) | 1 | 0.4 (0.16-1.02) | 1.3 (1.04-1.63) | 0.020 | 3484 | 50 | 1253 |
| Amitriptyline or clomipramine | 2.74 (0.81-9.26) | 1 | 0.66 (0.12-3.74) | 1.46 (1.05-2.01) | 0.024 | 1524 | 18 | 668 |
| All analyses are adjusted for age at start of medication period, study wave, mood stabilizing treatment, and sex.  Abbreviations: poor metabolizer (PM), intermediate metabolizer (IM), intermediate+ metabolizer (IM+), extensive metabolizers (EM), extensive+ metabolizers (EM), and ultra-rapid metabolizers (UM)  *Not adjusted for study wave due to lack of outcomes in some waves. | | | | | | | | |

| Table s17. Association between CYP2C diplotypes and risk of mania within 3 months of treatment initiation using 4 months between dispenses do define continuous treatment periods | | | | | | | | |
| --- | --- | --- | --- | --- | --- | --- | --- | --- |
|  | Slow^1^ | EM^2^ | Fast^3^ | HR for trend | P-value for trend | n | Events | Unique individuals, n |
| Escitalopram or citalpram | 1.26 (0.58-2.71) | 1 | 0.74 (0.35-1.58) | 1.1 (0.97-1.25) | 0.148 | 5487 | 54 | 1846 |
| Sertraline* | 1.79 (0.76-4.24) | 1 | 0.91 (0.39-2.13) | 1.11 (0.97-1.26) | 0.122 | 3484 | 50 | 1253 |
| Amitriptyline or clomipramine | 1.91 (0.55-6.72) | 1 | 0.42 (0.08-2.16) | 1.26 (1.05-1.52) | 0.011 | 1521 | 18 | 668 |
| All analyses are adjusted for age at start of medication period, study wave, and sex.  Abbreviations:  *Not adjusted for study wave due to lack of outcomes in some waves.  ^1.^ CYP2C19null/ CYP2C19null, CYP2C19null/ CYP2C:CG or TA, CYP2C19*17/ CYP2C19null, and CYP2C:TG/ CYP2C19null  ^2.^ CYP2C:CG or TA/ CYP2C:CG or TA  ^3.^ CYP2C19*17 / CYP2C:CG or TA, CYP2C:TG / CYP2C:CG or TA, CYP2C19*17/ CYP2C19*17, CYP2C:TG/ CYP2C:TG, and CYP2C:TG/ CYP2C19*17 | | | | | | | | |

Figure S1. Flow chart of the participant selection in the analysis of switch to another antidepressant from escitalopram and citalopram.

Abbreviations: poor metabolizer (PM), intermediate metabolizer (IM), intermediate+ metabolizer (IM+), extensive metabolizers (EM), extensive+ metabolizers (EM), and ultra-rapid metabolizers (UM), number of unique individuals (nid)

**

Figure s2. Flow chart of the participant selection in the analysis of switch to another antidepressant from sertraline.

Abbreviations: poor metabolizer (PM), intermediate metabolizer (IM), intermediate+ metabolizer (IM+), extensive metabolizers (EM), extensive+ metabolizers (EM), and ultra-rapid metabolizers (UM), number of unique individuals (nid)


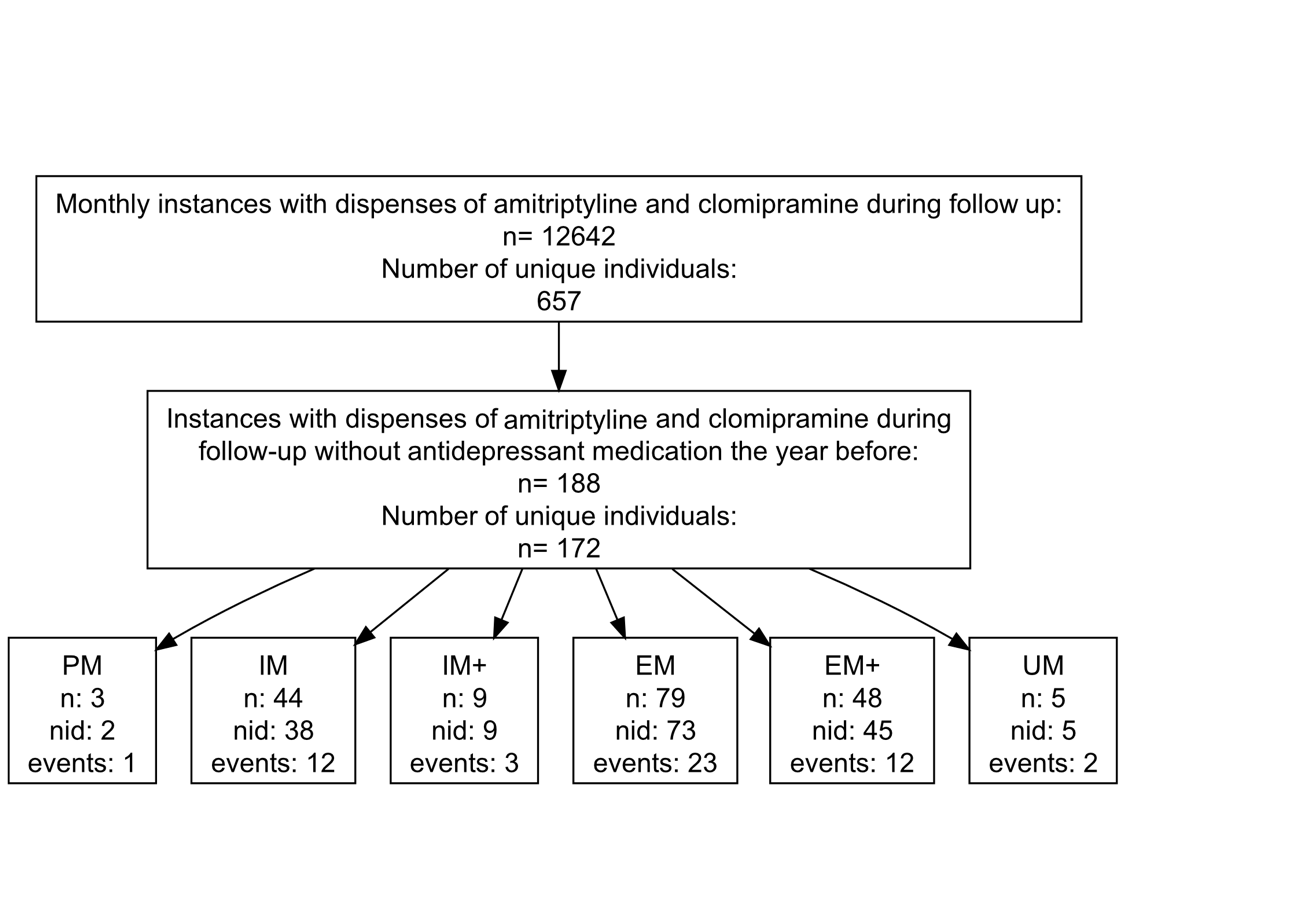


Figure s3. Flow chart of the participant selection in the analysis of switch to another antidepressant from clomipramine and amitriptyline.

Abbreviations: poor metabolizer (PM), intermediate metabolizer (IM), intermediate+ metabolizer (IM+), extensive metabolizers (EM), extensive+ metabolizers (EM), and ultra-rapid metabolizers (UM), number of unique individuals (nid)
